# Building Regional Threat-Based Networks for Estuaries in the Western United States

**DOI:** 10.1371/journal.pone.0017407

**Published:** 2011-02-28

**Authors:** Matthew S. Merrifield, Ellen Hines, Xiaohang Liu, Michael W. Beck

**Affiliations:** 1 Department of Science and Planning, The Nature Conservancy, San Francisco, California, United States of America; 2 Department of Geography and Human Environmental Studies, San Francisco State University, San Francisco, California, United States of America; 3 Global Marine Initiative, The Nature Conservancy, Santa Cruz, California, United States of America; National Institute of Water & Atmospheric Research, New Zealand

## Abstract

Estuaries are ecologically and economically valuable and have been highly degraded from both land and sea. Estuarine habitats in the coastal zone are under pressure from a range of human activities. In the United States and elsewhere, very few conservation plans focused on estuaries are regional in scope; fewer still address threats to estuary long term viability.We have compiled basic information about the spatial extent of threats to identify commonalities. To do this we classify estuaries into hierarchical networks that share similar threat characteristics using a spatial database (geodatabase) of threats to estuaries from land and sea in the western U.S.Our results show that very few estuaries in this region (16%) have no or minimal stresses from anthropogenic activity. Additionally, one quarter (25%) of all estuaries in this study have moderate levels of all threats. The small number of un-threatened estuaries is likely not representative of the ecological variability in the region and will require working to abate threats at others. We think the identification of these estuary groups can foster sharing best practices and coordination of conservation activities amongst estuaries in any geography.

## Introduction

Temperate estuaries are ecologically and economically valuable providing numerous critical ecosystem services, including nutrient cycling, nurseries for commercially important species and buffering against sea level rise [1], [2], [3], [4], [5], [6]. Coastal marine habitats, of which estuaries are a significant component, are estimated to provide over U.S. $14 trillion worth of ecosystem goods and services in the form of food, raw materials, disturbance regulation and nutrient cycling [7]. Despite these values estuaries continue to be degraded with most facing threats to their viability from both land and sea [8], [9], [10].

Multiple stresses including habitat alteration, nutrients, pollution, and sediment can be traced back to ever increasing demands for freshwater, food, timber, transportation, recreation, and waste disposal in their upstream watersheds [11]. These upstream activities eventually impact estuaries [12], [13], [14]. Nutrients such as phosphorus and nitrogen can be altered from natural levels by erosion, run-off from fertilized urban and agricultural land, and discharges from sewage treatment plants [15], [16]. Destructive silvicultural practices likeclear cutting reduces the capacity for soil to absorb precipitation and can increase the amount of sediment delivered to estuaries [17], [18]. Infrastructure for vessel trafficlike dredging, marinas, shoreline armoring, permanent opening of estuary mouth can alter hydrologic processes [19]. Dams impact habitat for anadramous fishes as well as the timing and quantity of freshwater and sediment inflow to an estuary [20]. Large scale development of aquaculture can impact estuary health with the release of non-native species, increased nutrients, waste, and habitat alteration [21]. Habitat alteration and destruction is along the west coast of the U.S. has resulted in some areas having lost over 90% of estuarine marshes and 99% of native shellfish beds [22], [23], [24], [25]. In Europe, less than 15% of the coastline is considered in ‘good’ condition. Some 22,000 kilometers of the European coastal zone are covered by concrete or asphalt and artificial surfaces increased by almost 1900 kilometers between 1990 and 2000 [26].

While these issues seem ubiquitous, conservation efforts tend to treat each estuary as a single system with unique problems and make little or no effort to coordinate efforts across regional geographic scales. This approach is inefficient, given the potentially strong similarities in estuaries' basic ecology and stresses to their viability [8], [27], [28]. Despite decades spent trying to protect estuary habitats, their health has continued to decline.

We could bolster estuarine conservation if we could identify more effective actions across estuaries in addition to those within them that are informed by a regional context. Currently there is little understanding of the spatial distribution of threats and their potential impacts to estuaries over large geographic areas [29]. Often conservation actions and decisions take place at local scales, without reference to the larger context in which they reside; this creates a need for more examples of regional systematic conservation planning devoted to estuaries [30], [31]. In addition**,** the geographic link estuaries provide between marine and terrestrial ecosystems creates a compelling setting to integrate these realms in conservation planning [32]. Restoration ecologists have stated the need for a coordinated strategy [33], and the Governors of California, Oregon and Washington have created an initiative acknowledging that regional cooperation is necessary for the development of conservation strategies at bays and estuaries [34].

Coordination will be difficult. Estuarine ecosystems are complex and present many challenges to integrate management ofecological and social dimensions present at any one place [35]. Addressing this problem will require collaboration by a diverse set of stakeholders at various organizational levels. Social networks can often develop to serve this purpose, enabling various actors to collaborate, share information and coordinate management efforts [36]. For example, evidence indicates that there were greater efficiencies in collaboration among estuaries within the U.S. National Estuary Program as compared to those outside the program [37].

The statusof digitalmapping efforts makes accounting of threats to estuaries over large regional geographies highly feasible, particularly in the coastal zone of the United States and other areas including Australia, New Zealand, and Europe.Once threats are accounted for at estuaries, they can be classified into groups that highlight commonalities. Classification efforts are a common approach to define representative groups in conservation planning [31], [38], [39], [40] however these are often ecological classifications based on biophysical data [as in 30]. Although the intersection of threats and biophysical types of estuaries has been addressed [41] none have explicitly accounted for threats at estuaries using similar methods.

**Figure 1:**
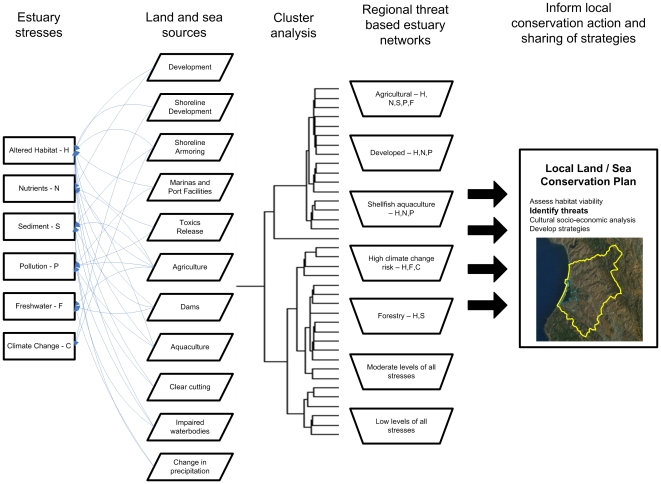
Conceptual diagram showing estuary stresses mapped from land and sea sources combined using cluster analysis to create regional networks. These networks can ideally inform local conservation actions.

To address the lack of regional threat information at estuaries, we identify multiple stresses to estuaries in the western United States, map the sources of these stresses (threats) from both land and sea for each estuary, and combine these estuaries into explicit groups to identify commonalities. The identification of these estuary groups is the first step towards building social networks that link geographically disparate estuaries based on common themes of threats.We anticipate that informing local groups about how what issues cross numerous estuaries can lay the groundwork to abate threats more efficiently through learning of shared experiences and common approaches (Figure 1).

## Methods

**Figure 2:**
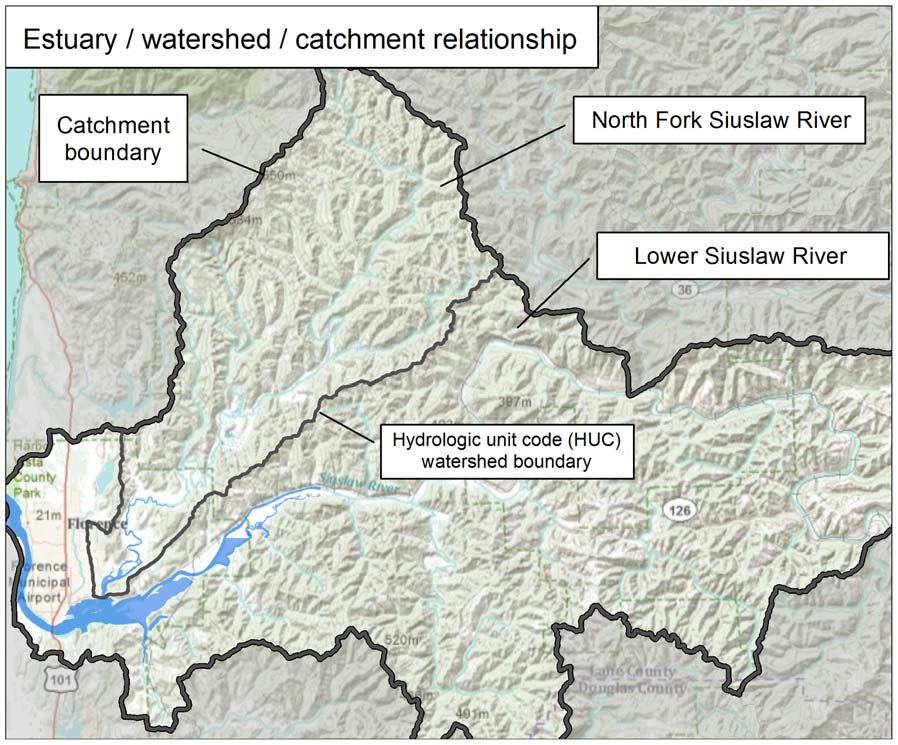
Map illustrating the relationship between estuary, watershed, and catchment boundaries at the Siuslaw River in Oregon. Catchments are made up of hydrologic unit code (HUC) 12 watersheds that are immediately adjacent to the estuary boundary.

We used a geographic information system (GIS) to map threatsat estuaries from both land and sea along the coasts of Washington, Oregon, and California (Figure 2). We sampled142estuaries, evenly distributed along the coast thatinclude a wide range of ecological conditions and high variability in sizeand structure (x = 4,101 ha, sd = 18,098 ha). All estuaries in the study are part of theCalifornia current large marine ecosystem and contain both Mediterranean and temperate coniferous forest ecoregionson land.

We identified six stresses to estuaries in the region: habitat alteration, nutrients, sediment, pollution, alteration of freshwater input, and climate change. Stresses are defined as physical, chemical and biological components of the environment that, when altered from their natural range of variability by human or other activities, can result in degradation to estuarine ecosystems. For example, agriculture in the watersheds upstream of estuaries can be one source of increased nutrient stress. The sources of each stress are referred to as threats and were mapped using readily available spatial data (Table 1). Relative amounts of each threatin estuaries and their upstream watersheds were calculated (e.g. percent of watershed in urban, density of dams in watershed) to create threat variables.These variables capture a range of human impacts at estuaries and are notexhaustive. In all cases variableswere considered only if there was readily available spatial data mapped with similar methodology across the entire study area.These data represent the current condition of estuaries and do not explicitly account for historical impacts.Ideally we would characterize not just current but also historical impacts. Indeed these legacy impacts surely have had real repercussions at estuaries. Unfortunately most of the evidence is patchy and anecdotal and cannot be compiled in regional analysis. Moreover, the current stressors are the ones we can readily address.

**Table 1:** Estuary stresses.

Stress	Land and sea source	Spatial data source
Hab alt, nutrients, pollution	Development in catchment	NOAA C-CAP
Hab alt, climate change, sediment	Development of estuary shoreline	NOAA C-CAP
Hab alt, climate change, sediment	Shoreline armoring in estuary	NOAA ESI
Hab alt, pollution	Port facilities in estuary	USACE, NOAA ESI
Nutrients, pollution	Toxics release in catchment	EPA NPDES/TRI
Hab alt, nutrients, pollution	Agriculture in catchment	NOAA C-CAP
Hab alt, freshwater	Dams in catchment	WA DOE, OR WRD, PSFMC
Hab alt	Approved waters for shellfishing	NOAA CA&DS
Sediment	Clearcutting in catchment	NOAA C-CAP
Nutrients, pollution, sediment	Impaired waterways in catchment	Clean Water Act (303d)
Climate change, freshwater	Change in future precipitation	Hadley Centre HadCM3

*Key to acronyms: NOAA C-CAP/CA&DS/ESI, National Oceanic and Atmospheric Administration Coastal Change and Analysis Program/Coastal Assessment and Data Synthesis/Environmental Sensitivity Index. EPA NPDES/TRI, U.S. Environmental Protection Agency National Pollutant Discharge Elimination System/Toxics Release Inventory. WA DOE, Washington Dept. of Ecology, OR WRD, Oregon Water Resources District, PSFMC, Pacific States Fishery Management Council USACE, US Army Corps of Engineers.*

Estuary stresses, their land or sea source, and the spatial database used to map them. Sources (threats) were mapped and summarized for every estuary in the study region. Note: Hab alt  =  Habitat alteration.

All threat variables were combined into a single matrix to support a statistical classification of the estuaries. Hierarchical cluster analysis was used to classify estuaries into logical networks, which share similar amounts of values for all variables (Figure 1). Mean variable values for each network were examined to determine the predominant threat for the network. (Table 2). Summary geographic statistics (total and average size, standard distance, percent sample) highlight the geographic extent of each threat (Table 3).

**Table 2:** Mean values for threat variables for each network (development, shoreline development and armoring, port facilities, toxics release, agriculture, dams, shellfish aquaculture, clearcutting, 303(d) streams, precipitation reduction).

Network	Dev. %	Shr Dev. %	Shr. Armm/ha	Port facilities.#/ha	TRI #/ha	Agri. %	Dams#/ha	Shellfish Aqcltr %	Clear cutting %	303d Str. m/ha	Precipreduct. %
1	20.7	19.8	0.3	0.3	5.6	56.6	0.3	0.0	0.0	34.3	19.4
2	71.4	52.6	2.2	9.4	29.7	4.6	2.2	0.0	0.0	13.2	7.4
3	11.3	29.2	0.8	3.4	1.6	7.2	2.6	1.6	0.2	7.0	12.3
4	5.3	10.8	0.5	5.1	3.0	6.0	1.6	52.5	1.6	14.0	6.4
5	3.5	13.2	3.2	2.4	0.5	4.0	0.9	0.0	0.2	91.6	13.7
6	2.4	4.7	0.3	0.5	0.0	0.8	1.7	0.1	0.8	4.2	7.7
7	5.3	44.1	0.0	0.0	0.0	2.2	0.0	0.0	0.0	0.0	53.1
8	2.0	7.8	0.6	1.2	0.3	3.5	1.4	0.0	2.0	46.5	9.2
9	2.0	8.5	0.1	0.3	0.0	0.2	0.4	4.8	5.6	2.9	3.6

High values for any threat indicates relatively more stress on the estuary.

**Table 3.** Geographic summary statistics for each estuary network.

Network	Primary Source of Stress	Number	Std. dist (km)	Sum area (ha)	Avg area (ha)	Propn. area	Propn. Sample
1	Agriculture	6	153	1,837	306	0.3%	4.3%
2	Development	22	103	9,222	419	1.6%	15.7%
3	All (moderate levels)	35	608	378,638	10,818	65.0%	25.0%
4	Shellfish aquaculture	19	454	138,247	7,276	23.7%	13.6%
5,8,9	Forestry/water qual.	30	411	47,881	1,596	8.2%	21.4%
6	Few (low levels)	23	518	6,631	288	1.1%	16.4%
7	Climate change	5	8	10	2	0.0%	3.6%

Standard distance is a measure of the spatial dispersion of the network, lower values are compact, higher values are spread out. The proportion area is the total area of the network divided by the total area of all estuaries in the study. Proportion sample is the number of estuaries in the network divided by the total number in the study.

### GeographicData

For coastal areas of the western United States,geospatial data mapped at a common scale are readily available, providing an efficient source of information for this analysis (e.g. NOAA Environmental Sensitivity Index, Coastal Change and Analysis Program). We used a geographic database, or geodatabase, to storedigital information on thespatial distribution of each variable. Two geographic units were used to analyze spatial data for each threat: estuaries and catchments.Here we define a catchment as the aggregation of upstream watersheds that contribute surface runoff to the estuary (see methods below). The two units allow us to accommodate the different spatial extents of each threat. For example, values for shoreline armoring and port facilities were summarized at the estuary unit, while values for agriculture, development and dams, were aggregated at the catchment unit. Every estuary was assigned one or more catchments such that all variables could be aggregated to any single estuary.

Estuary boundaries were compiled from various digital sources. The National Wetlands Inventory (NWI) mapped a majority of estuaries in this study. In areas where the NWI is incomplete, estuary boundaries were inferred by the presence of salt marsh or tidal flats mapped by shoreline segments of NOAA's Environmental Sensitivity Index (ESI). We placed more emphasis on capturing the presence of an estuary rather than focusing on the details of its boundary and think this is appropriate given the regional scale of this study.

To explicitly integrate land based threats with estuaries, we created an analytical unit composed of adjacent upland watershed boundaries we term catchments. Catchments areaggregations of watersheds immediately adjacent to the estuary boundary. We used watershedsfrom the Watershed Boundary Dataset, which divides areas into successively smaller hydrologic units. Each unit is identified by a unique hydrologic unit code (HUC). We delineated catchments using the finest scale watersheds, referred to as HUC12, that were immediately adjacent to the estuary boundary (Figure 2).

After the estuaries and catchments were mapped, spatial data for each threat werecompiled and assigned to the estuary or catchment using a GIS overlay. Each threat was formulated to specifically address the limitations or assets of the spatial data used to represent that threat. Given the variability in area of catchments within the region, relative as opposed to absolute values were summarized for each catchment by dividing the sum total of the threat variable by the total area of the catchment or estuary. Below are descriptions of each threat variable and the GIS procedures used to compile the data.

Agricultural and urban land useswere extracted from the NOAA Coastal Change and Analysis Program (C-CAP) database.Agriculture includes cultivated land, pastures and hay. Proportions of urban and agricultural land use were calculated as percent of each type in the associated estuary catchment.

Point sources of nutrients and pollution were mapped from industrial facilities, ports, and marinas.We used the Toxics Release Inventory (TRI), whichis a spatial database of chemical releases and waste management activities regulated by the U.S. federal government. The number of TRI sites per unit catchment area was calculated as a proxy for point source pollution and nutrient inputs. Similarly, port facilities such as wharfs, marinas, and ferry landings weremapped as point locationswith densities calculated per unit area of the estuary.

We also examined issues of water quality and pressures from bottom harvesting and aquaculture by calculating the estuary area within approved and conditionally approved shellfish growing areas using data from the 1995 National Shellfish Register. Bays with higher amounts of approved waters are indicative of areas with better water quality but also likely higher harvesting and aquaculture pressure. It is important to note that outside the U.S., growing areas refer to active aquaculture and harvesting locations, whereas our dataset indicates the suitability for aquaculture at a given estuary.

Pollution and sediment stresses were also mapped with impaired waterways as listed bysection 303(d) of the U.S.Clean Water Act which requires states to identify waterways that do not meet federal water quality standards. These waterways represent various sources of pollution delivered to estuaries. Impaired waterway lines were intersected with the catchment boundaries to calculate thelinear density (km/ha) for the catchment.In addition to impaired waterways, clear cutting within the catchment was mapped as a source of sediment stress. Using the C-CAP database, clear cuts were defined as areas that went from mixed or evergreen forest to bare land or grassland between 1995 and 2000. The clearcut threat was calculated as the proportion of the catchment with clearcut areas.

Shoreline armoring (riprap, seawalls) and infrastructure (roads, buildings) along the margins of estuaries indirectly measure risk to sea level rise by creating barriers to upland migration for adjacent coastal marsh habitats. Additionally shoreline alterations reduce habitat and alter sediment flow within the estuary.We mapped these threats using two methods: 1) theproportion of urban land use within a 100 m buffer of the estuary using the C-CAP database, and 2) the linear density of artificial shoreline per unit length of the estuary usingriprap and man-made mapped by NOAA's Environmental Sensitivity Index (ESI) database.

As a measure of freshwater and habitat alteration, we calculated the number of dams per unit catchment area.Locations of dams were compiled from various state sources. Attributes such as dam height and storage capacity were not common to all databases; therefore all dams were treated equally regardless of size or capacity.

Change in future climate mayalter precipitation patterns which in turn would alter freshwater inflow to estuaries.To account for this stress, we used an end of the century (2070–2099) annual precipitation change projection from the Hadley Centre's HadCM3 model with a business as usual emission scenario (after IPCC 2007). Changes in precipitation were normalized against the current observed climatology from 1960–1990 provided by WorldClim [42]. Predicted change in future precipitation was calculated using Equation 1:
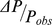
(1)


Where 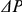 is the projected futurechange in average annual precipitation and 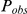 is current observed climatology for each catchment.

### Statistical analysis

Estuaries were segmented into groups,or networks, with similar stresses and threatsusing elevenvariables (Table 1). Variables were formulated with similar directions(e.g.large amounts of development and a high density of dams in the catchmentboth equal high stress to an estuary). Values were standardized using their range to eliminate variation in units of measure [43]. Standardized data were then converted to a matrix of Euclidean distance between all variable values for every estuary in the study. Correlations between all variables were low, with the highest between shoreline development and development in the catchment (*r* = 0.68). To create estuary groups, we ran a cluster analysis on the distance matrix using R statistical software [44]. The cluster analysis reduces *n* original estuaries into *g* groups such that *1*<*g*<*n* with the general goal of minimizing within-group variation and maximizing between-group variation.

There was nothing to suggest the number of groups *a priori* so we chose hierarchical agglomerative clustering with Ward's method. To determine the optimal number of groups and the robustness of group membership, we explored k-means partitioning and a maximum likelihood classification. K-means partitioning measures differences in cluster groups by their variance (sum of squares). Plotting the number of groups by the within group sum of squares reveals an inflection pointat the optimal number of groups. The maximum likelihood classifier applies a number of geometric models to minimize within group variability. The maximum likelihood classifier identifies an optimal set of groups using a model of ellipsoids with equal volume and equal shape to define cluster membership. All three methods (hierarchical, k-means, maximum likelihood) derived approximately the same number of groups with similar variable composition (e.g. highly developed) and members. We reviewed estuary group members by visualizing spatial data onvariableswith other contextual information (e.g. digital aerial imagery) to determine if membership made intuitive sense (e.g. southern California estuaries in the “developed” group). Each group is subsequently referred to as a network.

## Results

**Figure 3:**
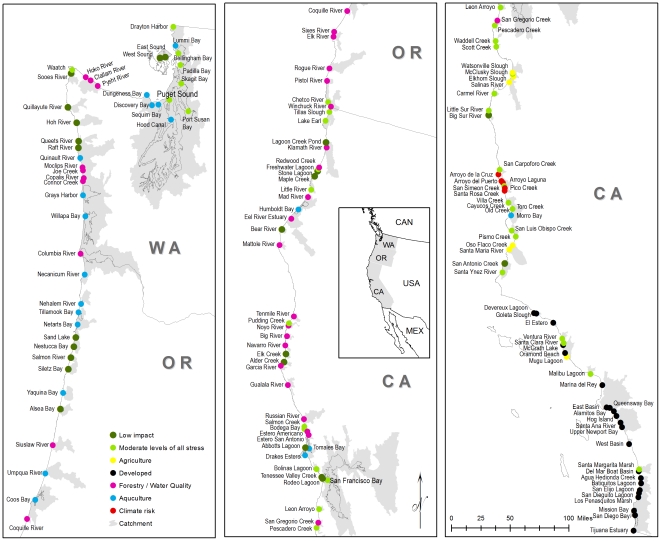
Map of estuary networks in the study region. Networks were created using hierarchical cluster analysis of 11 variables that represent stresses to estuaries in the region.

**Figure 4.**
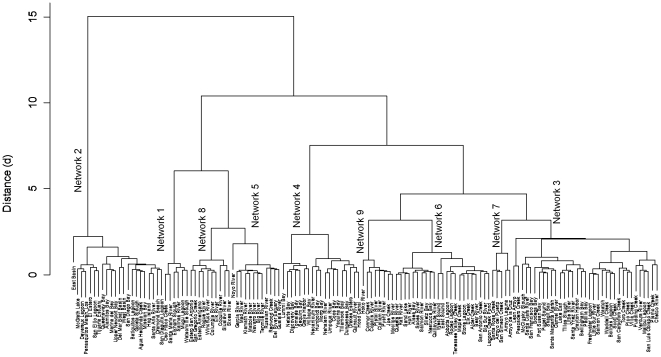
Dendrogram showing hierarchical division of estuary networks and agglomeration schedule. The hierarchical clustering identified nine substantive networks at a Euclidean distance of 2.5. Major divisions are by development (Network 2; d = 16), impaired inflows (Networks 1, 5, 8; d = 10), and approved shellfish growing areas (Networks 4; d = 7.5).

The hierarchical clustering identified nine substantive networks illustrated by the map in Figure 3 and the dendrogram in Figure 4. The properties of estuaries within the networkwere described by variable means (Table 2). Geographic summary statistics for each showed the distribution and magnitude of each network (Table 3). Close investigation of these tables andthe dendrogram reveal major divisions by development (Network 2; d = 16), impaired waterways (Networks 1, 5, 8; d = 10), and estuaries with substantial areas approved for shellfish and aquaculture (Network 4; d = 7.5). The remaining Networks: 3, 6, 7 and 9 are moderately impacted by single threats. Network 6 represents the overall lowest impact estuaries; however they cover a small proportion of the sample and area. Network 3 has low to moderate levels of all threats.

Six estuariesare in network 1, accounting for 4% of the total number of estuaries and 0.3% of the total area withan average 56% of their catchment in agricultural land use. These estuaries are entirely within central California (Point Conception to S.F.Bay) and have a moderate climate risk (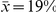 reduction in precipitation by 2099 according to HadCM3). Network 1 estuaries also have a moderate to high amount of EPA 303(d) streams, which may be attributed to the agriculture. Examples from this network are Elkhorn Slough, and the Salinas and Santa Maria Rivers in California.(Figure 3).

Estuaries in network 2 have heavily urbanized catchments, with 71% of their area on average in urban or developed land use. These are 16% of the total number of estuaries and 1.6% of total estuary area. These 22 estuaries are concentrated entirely within southern California (Point Conception to U.S./Mexico border). Along with urbanization comes a large amount of shoreline armoring, port facilities, and toxic release sites. This network includes major industrial ports such as Long Beach Harbor and San Diego Bay and represents some of the most impacted and heavily utilized estuaries in the study area.

Network 3 has moderate levels of all threats. These 35 estuariesare 65% of the sampleand 25% of the total area making it one of the largest networks. The inclusion ofSan Francisco Bay and Puget Sound in this network accounts for the large overall area. These estuaries are geographically disperse, with a singlegeographic outlier, Santa Margarita Marsh in southern California. A majority of these estuaries have reasonably intact catchments (e.g. Bolinas Lagoon, Scott Creek) but are bounded by either roads or some type of urban infrastructure making them susceptible to sea-level rise. These estuariesare also under a moderate climate risk, and some like San Carpaforo Creek in the central coast of California (San Luis Obispo Co.), could experience as much as a 25% reduction in precipitation by 2099. Two estuaries from this group (Leon Arroyo and Pescadero Creek, both in San Mateo Co. CA.) have the highest density of dams in their catchments.

Estuaries in network 4 have a high proportion of their waters approved for shellfish harvest and aquaculture. This is the second largest network representing 13% of the sample and 23% of the total area. This network is geographically compactand largely contained within northern Oregon (CoosBay, YaquinaBay) and Washington (WillapaBay, Grays Harbor). The southern outliers are four California estuaries (Humboldt, Tomales, Drakes, and MorroBays). These estuaries havelow tomoderate levels of all other issues and threats with somehigh amounts of impaired waterways (Humboldt Bay, CA, Necanicum River, OR.).

The estuaries in groups 5 and 8both have high proportions of impaired waterways in their catchments. Group 9 has a relatively high amount of clear cutting. We combined groups 5, 8 and 9 into a single network representing incompatible forestry and water quality threats. Together they make up 21% of the sample and 8% of total estuary area. These estuaries have some of the highest amounts of clearcutting in their catchments (Hoko, Clallam, and Pysht Rivers in Washington). Two estuaries in this network (Estero Americano and San Antonio in Marin Co. California) are geographic outliers and their high proportion of sediment ismay be related toerosion from livestock operations.

Network 6 represents the estuaries with the least threats. These 23 estuaries make up 16% of the sample and1.1% of total estuary area. Their distribution of size is similar to the entire sample but does not include any of the larger estuaries (>2,000 ha). These estuaries are largely north of San Francisco Bay with geographic clusters in central Oregon (Salmon River, Sand Lake, Siletz Bay, Nestucca Bay) and two outliers in the south, the Big Sur River and San Antonio Creek in Monterey County California). Network 7 is similarly low impact but represents the highest risk of reduced precipitation in the entire region (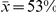 reduction in precipitation by 2099according to HadCM3). These five estuaries are relegated to a small geographic envelope on the California central coast.

## Discussion

There are few if any coastlines where estuaries have been managed on a regional basis. Consequently, conservation and management for estuaries in the U.S. and elsewhereaddressed on a bay-by-bay basis [29]. This is in contrast to offshore systems which are often addressed in regional programs (e.g. Regional Seas Programmes, Large Marine Ecosystems, Regional Fishery Management Councils). The combination of impacts along the land-sea interface creates unique conservation and management challenges, making a good case for planning that integrates marine and terrestrial processes [32], [45]. This paperattempts to address both these issues by 1) accounting for threats to estuary viability from both land and sea in a spatially explicit framework and 2) defining thematic networks of estuaries based on predominant threats. The results provide regional context for local management and a framework for future collaboration and learning.

Management of ocean and coastal resources is moving towards more regional scale efforts in Europe, China, Canada, Australia, the U.S. and elsewhere [46]. There has been increasing interest in the U.S. and around the world in ecosystem based management of coastal and marine resources which requires a coordinated approach [47], [48], [49]. Subsequently regional ocean governance is being addressed by partnerships that focus on coastal and marine spatial planning (CMSP) as a means to support integrated management of resources. The type of spatially explicit information and analysis herein could be useful to CMSP efforts in many geographies and help in development of regional approaches across estuaries in the coastal zone. We expect this to help in the development of policies that will transcend individual estuaries and ideally provide more comprehensive actions and conservation outcomes.

Our results identify seven threat-based networks of estuaries in the region (Table 3). However, distilling those networks into three principle categories is helpful for interpretation: 1) estuaries with minimal or no threats, 2) estuaries that have a single dominant threat and 3) estuaries that have some level of all threats. These categories draw attention to the distribution of threats in the region and provide insight into potential conservation opportunities and strategies at multiple estuaries. Below we discuss the significance and potential of each.

### Estuaries with minimal or no threats

A primary concern is the relatively small number and size of estuaries that have none or low levels of all threats(network 6). Their small size and distance from major urban centers make them unattractive for development; often having large amounts of managed lands in their catchments, 57% on average. This suggeststhat land protectionworks to abatethreats, however has only happened in rural estuaries. This network of estuaries provides a valuable lesson in addressing threats, maintaining ecological function, and effective management. Identifying them is the first step in sharing learned successes and failures; managers at these estuaries may benefit from sharing practices through a social network that is outside their realm of expertise and agency affiliation. The ability to export these lessons to other places grappling with similar management issues can foster information and relationships similar to the National Estuary Program [37]. This network also brings to light the heavy impact to southern California estuaries discussed below.

### Estuaries with a single dominant threat

Future efforts at impacted estuaries will be valuable to ensure overall representation of variability. Broadening the scope of estuary conservation will require expanding effort into adjacent networks with manageable threats. A majority of estuaries in the region (59%) are dealing with single threats and issues. Two of those networks, those with forestry and substantial areas approved forshellfish andaquaculture, present interesting cases.

Estuaries dominated by watersapproved for shellfish aquaculture and harvest are some of the larger estuaries in the region (7,276 ha on average) covering 23% of the study area. They are concentrated in northern Oregon and Washington with a few examples in California (Figure 3). The approved designation for shellfish harvest determineswhetherthe estuary can support edible shellfish and therefore a indication of good water quality. Indeed in areas where there are active aquaculture interests, there are strong advocates for measuring and maintaining good water quality. As a result,the shellfish harvest and aquaculture industryshare similar objectives with conservation organizationsin minimizing incompatible land use upstream. Harvesting and aquaculture can impact estuarine ecology particularly through bottom disturbance and in the past (at least in the U.S.) with the introduction of non-native invasive species [21]. In all cases these estuaries have relatively low levels of other issues and threats. Coalitions between business (aquaculture and fishing) and conservation interests represent an important strategy for maintaining and improving conditions in these estuaries [50].

Estuaries that have catchments dominated by incompatible forestry (e.g. clear cutting) may represent another conservation opportunity in the region. Incompatible forestry in estuary catchments can increase sediment loads; however, unlike development or agriculture, forest landscapes are dynamic and have potential for good management in the future. Networks 5, 8 and 9 have high proportion of clear cutting and impaired waterways in their catchments. Those with the highest proportion of impaired waterways occur entirely within northern California. Closer investigation of the listed waterways in these catchments reveals that sedimentation and siltation are listed as the predominantpollutant stressors, followed closely by temperaturewhich can indication of reduced canopy cover over stream reaches from forestry [17], [51] (Table 4). Half of these estuaries are in California and private timber companies regulated by the California Forest Practice Rules dominate their catchments. The other half, in Oregon and Washington, are dominated by National Forests which contribute to60% of timber productionin these states [52]. The estuaries in this network present anopportunity to engage on forest policyat federal and state levels. Currently a memorandum of agreement between the U.S. Environmental Protection Agency and the U.S. Forest Service focused onwater quality is a step in that direction. Measuring the impact of policy on conservation outcomes can be difficult; however this analysis allows us to determine how many estuaries could be affected by forest policy changes.

**Table 4:** Frequency of pollutants listed by section 303(d) of the Clean Water Act for catchments in networks 5, 8, and 9.

303(d) pollutant	Count
Sedimentation/siltation	192
Temperature	96
Nutrients	16
Sediment	15
Organic enrichment/dissolved oxygen	13
Others	6

The counts of sediment/siltation and temperature are likely due to incompatible forestry.

Other single issue estuaries may be more difficult to address, particularly those dominated by development and agriculture. The highly urbanized estuaries, almost entirely in southern California, emphasize the need for working in that area. The question for these places is one of priorities; should restoration focus on relatively intact systemsor restoringheavily impacted estuaries with small amounts of fragmented habitat. Additionally, atmospheric deposition from transportation and industry as well as municipal wastewater disposal contributes high amounts of nitrogen to these estuaries. Given their proximity to large numbers of people, projects in these estuaries can educate the proximate public about theservices they provide in the way of nutrient cycling and storm surge buffer.

Agriculture dominated estuaries present significant challenges. Humans are supported by an agricultural system that relies on synthesizing vast quantities of nitrogen that escape from agricultural landscapes and transported to estuaries through rivers and groundwater [16]. Only a few estuaries in the study area (n = 6 or 4% of total) have catchments dominated by agriculture andthey mostlyoccur entirely in central California. Given the large revenues generated by agriculture from places like the Salinas River Valley in California and the Nooksack River in Washington, a full scale shift in agricultural practice is not likely. Although riparian vegetation can absorb excessive nutrients so agricultural setbacks from streams and restoration of riparian habitat in these catchments would be important. Knowing the regional impact of estuaries can inform other governmental and non-governmental organizations to set quantitative restoration goals within the region, similar to efforts in Chesapeake Bay. Additionally sharing information about successful management at these estuaries can broaden the scope of best practice in the region.

A small set of estuaries in central California (network 7) have a high risk of reduced precipitation in the future. These small coastal streams and lagoons currently have limited amounts of freshwater inflow from local water use and current climate conditions. Future reductions in precipitation will only exacerbate any issues related to freshwater delivery to these estuaries. Even though we only used a single model to predict climate change, management and planning efforts inestuaries that consider changes in freshwater would be prudent. Applying the results of regional climate models to local scale phenomenon is problematic; therefore any consideration of future climate should be analyzed at a local scale. This study provides a first look at the scope of the problem and may serve to prioritize climate adaptation efforts to high risk estuaries.

### Considerations for estuaries with multiple threats

Managing their existing threats and minimizing future impact is crucial to long term viability of estuaries. The presence of multiple threats at estuaries raises the issue of synergy. Further research should consider the compounding or confounding roles of multiple threats in estuaries to determine appropriate strategies. Our database can support further parsing of these multi-threat estuaries to determine which combinations show the highest impact. Below we discuss the implications of multiple threats within some estuary networks.

Agriculture dominated estuaries (network 1) highlight the compounding effect of future climate change. The HadCM3 model predicts a 19% reduction in precipitation on average in these estuaries by 2099. This reduction, combined with freshwater drawdown from agriculture creates a problem for estuary residence time and salinity, both of which drive the cycle of primary production. Strategies at estuaries dealing with large amounts of agriculture should address not only nutrient absorption, but also the securing of sufficient freshwater supply in the future. Any additional efforts (e.g. dam removal, timing of drawdown) will be reasonable adaptation efforts in these estuaries.

The highly developed nature of southern California highlights the need to focus on geographic outliers, calling attention to the largest network in the study. One quarter of all estuaries in the region, representing 65% of total area, have moderate to low levels of all threats. These occur throughout the region; however examples in southern California (Ventura and Santa Clara Rivers, Malibu Lagoon, and Santa Margarita Marsh) may represent opportunities to maintain representative samples in this part of the region (Figure 3).

### Limitations

The regional scope of this paper provides consistent information over a large set of estuaries; however local situational investigations of political, cultural and socio- economic context are necessary.Any plan to minimize threats at estuaries must involvethe local stakeholders responsible for creating or abating them. Expanding the scope of threat-based networks to include these social dimensions is not only necessary but will facilitate sharing ideas. We think that identifying threat-based networks can serve as a catalyst to build stronger social-ecological relationships that go beyond individual estuaries.

Threats are only one dimension of determining the long term viability of estuaries. How these threats interact with the physical environment is crucial in establishing strategies to abate them. For example, estuarine networks that are dominated nutrient and pollutant stresses could be mitigated by presence of riparian and estuarine habitats. Additionally, pairing the threat networks with a biophysical classification (e.g. size, shape, temperature, salinity) can inform the assumption that stresses to estuaries are affected by their physical composition and make for a more robust evaluation of estuaries in the region [41].

In any geographic analysis, scale of available data and the assumptions about results derived from those data are important. Threats occur at multiple spatial scales and their location at finer resolutions may alter their impact. For example, some estuaries suffer from sediment scouring due to the location of the armoring relative to the mouth.Some indication of armoring proximity to mouth or head of the estuary may add a beneficial nuance and a better estimate of this threat. Finally, the inclusion of mega-estuaries in the study (San Francisco Bay, Puget Sound and Columbia River) are likely placing them in networks that do not accurately represent the complexity of their issues.

### Conclusion

Conservation planning in the coastal zone requires an assessment of both land and sea based threats. This paper provides a spatially explicit picture of the predominant stresses to estuaries and identifies commonalities at a regional scale to articulatethreat-based networks. Our results indicate that few estuaries are unaffected by stress and the majority are subject to some level of all stresses. Representing a wide range of ecological variability in the region will require working beyondminimally impactedestuaries and managing those with multiple threats. There are good opportunities, as a majority of west coast estuaries are dominated by a single threat. Dealing with single threats at multiples sites, like incompatible forestry or working towards common water quality goals with the shellfish aquaculture industry, would make large conservation gains. These regional networks provide a picture of predominant threats shared across many estuaries, however all strategies on how to best abate threatsmust be locally informed. In depth assessment of the socio-economic and cultural context of each estuary and its contributing watersheds is essential. These results highlight two key pieces of information. First, a picture of the scope of estuary threats at a regional scale. Second this analysis identifies estuaries with common themes, grouping them into networksthat ideally facilitate information sharing. These two factors we think will advance priority setting for conservation action and serve as the basis for social networks that strive to share successes and failures of conservation action.This approach can be applied to other regions as a means of coordinating conservation activities and ideally moving beyond a bay by bay approach to conservation at estuaries.
